# Chinese and western medicine treatment of myocardial fibrosis drugs

**DOI:** 10.3389/fcvm.2024.1477601

**Published:** 2025-01-15

**Authors:** Yuxi Zhu, Fangmei Zhang, Zhongcheng Li, Yu Zhou, Yi Shu, Jian Ruan, Guo Chen

**Affiliations:** ^1^ Department of Acupuncture, Bao’an Authentic TCM Therapy Hospital, Shenzheng, China; ^2^ Graduate School, Jiangxi University of Chinese Medicine, Nanchang, China; ^3^ Fever Clinic, The 334 Affiliated Hospital of Nanchang University, Nanchang, China

**Keywords:** myocardial fibrosis, Western medicine, Chinese medicine, drugs, excessive extracellular matrix

## Abstract

Myocardial fibrosis (MF) is a common pathological manifestation of many cardiovascular diseases, such as myocardial infarction, myocardial ischemia, and sudden cardiac death. It is characterized by excessive proliferation and activation of fibroblasts, transformation into myofibroblasts, and, eventually, excessive deposition of the extracellular matrix, resulting in heart damage. Currently, modern drugs such as angiotensin-converting enzyme inhibitors, diuretics, and β-blockers can improve myocardial fibrosis in clinical treatment, but their therapeutic effect on this disease is limited, with obvious side effects and high cost. Traditional Chinese medicine (TCM) has the advantages of multiple targets, low cost, and few side effects. Traditional Chinese medicines, such as Salvia miltiorrhiza, Astragalus, and Angelica extracts, and patent Chinese medicines, such as Qiliqiangxin capsules, Shenqi Yiqi dropping pills, and Tongxinluo capsules, can improve myocardial fibrosis. In this review, current Chinese and Western medicine methods for treating myocardial fibrosis are discussed. The signaling pathways and targets of Chinese and Western medicine are involved in the treatment of myocardial fibrosis. This review aimed to provide valuable insights and ideas for both clinical treatment and basic research on myocardial fibrosis.

## Graphical Abstract

Figure 1 Graphical summary (Figure 1 created with Biorender.com).

## 1 Introduction


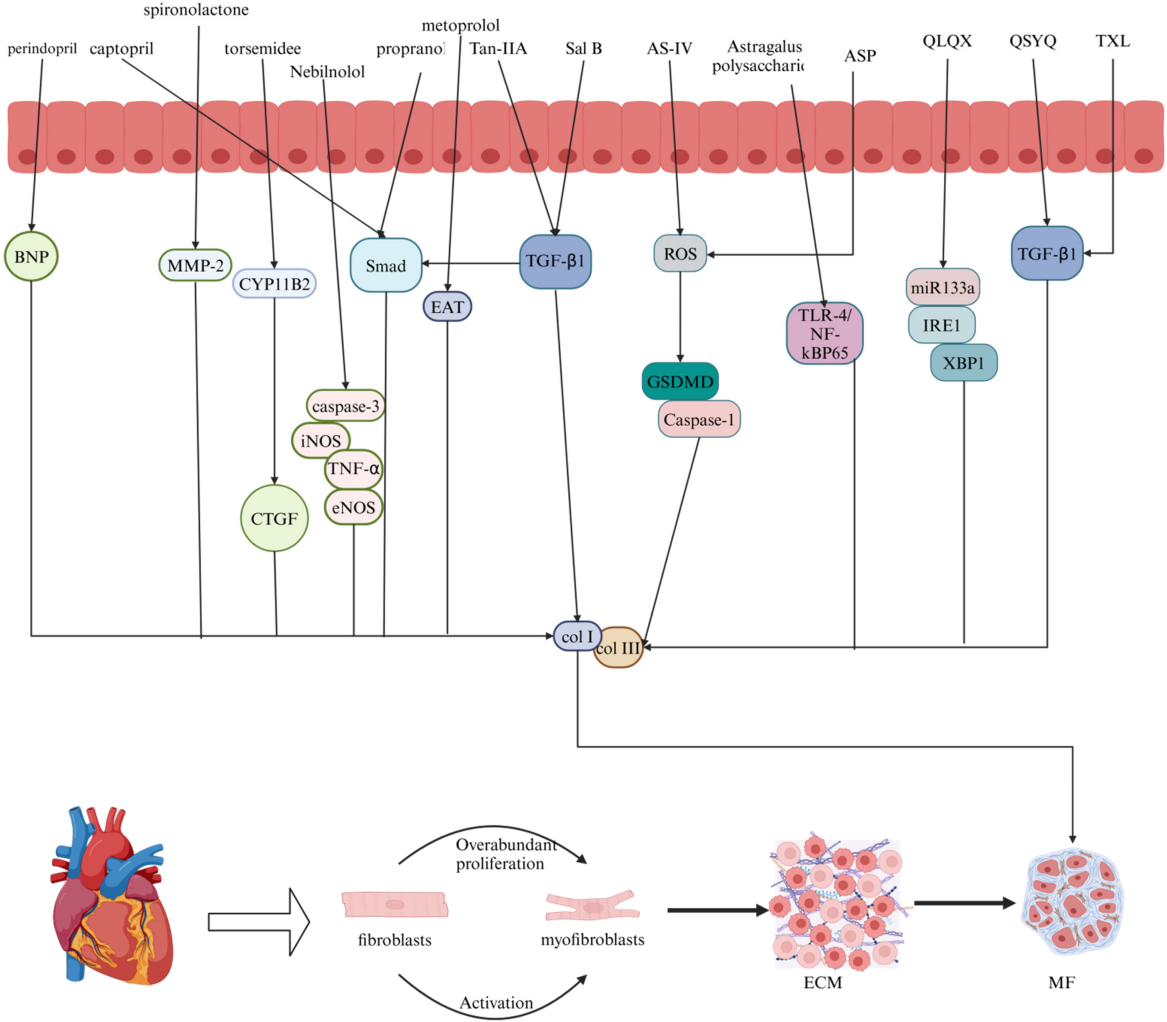


MF frequently appears as a pathological feature in a range of cardiovascular conditions, such as myocardial infarction, myocardial ischemia, and unexpected cardiac death (1). Following myocardial infarction, the accumulation of collagen I (Col I) and collagen III (Col III) leads to increased MF and the formation of non-contractile scar tissue. Additionally, myofibroblasts in the infarction scar contribute to excessive extracellular matrix (ECM) deposition, resulting in cardiac damage and eventual remodeling (2). When myocardial ischemia occurs, inflammatory cells activate the transforming growth factor-β receptor 1 (TGF-β1) pathway, leading to an increase in matrix protein accumulation and worsening of MF (2–4). In cases of sudden cardiac death, thickening of Col I and Col III within the heart tissue is observed, which leads to myocardial fibrosis and increased cardiac stiffness (5). Cardiovascular diseases affect millions of people globally, imposing a significant economic burden and ranking as a leading cause of human mortality (6). The main pathological characteristics of MF include the transformation and activation of cardiac fibroblasts (CFs) into myofibroblasts, the accumulation of excessive collagen, increased stiffness of the ECM, scar tissue formation, and structural and functional alterations in the heart, which ultimately lead to reduced cardiac function (7, 8). The mechanism of MF is intricate, with rapid onset and high mortality rates (9). Modern medicine offers limited therapeutic options for this disease with high treatment costs (1). As an alternative, traditional Chinese medicine is known for its multiple targets, minimal side effects, and low cost (10). Given the extensive research on MF treatment in traditional Chinese medicine, this article aims to review and summarize current advancements to serve as a foundation for future clinical and experimental studies (11).

When traditional Chinese medicinal materials are utilized for disease treatment, they are often subjected to multiple rounds of steaming and drying to enhance their therapeutic properties and mitigate potential toxicities and side effects (12). TCM has demonstrated promising outcomes, with a rich history of treating cardiovascular diseases (13, 14). Recent studies have increasingly indicated that traditional Chinese medicine could open novel avenues for MF treatment research (7). Owing to its diverse composition, ability to target multiple sites, and minimal adverse effects, traditional Chinese medicine allows its various chemical constituents to follow distinct therapeutic pathways, reaching multiple targets to address MF effectively (15). Nevertheless, the precise therapeutic mechanisms of traditional Chinese medicine remain unclear (16). Currently, most traditional Chinese medicine interventions are at the stage of animal experimentation, with limited clinical trials and reliance on singular research methodologies, resulting in a dearth of exploration of molecular mechanisms (17). Given the variability in patient symptoms, individualized syndrome differentiation and treatment are necessary, highlighting the lack of a standardized syndrome differentiation system (7). Furthermore, improvements in production techniques, processing methods, and environmental quality are needed to increase the efficacy of traditional Chinese medicine (18).

This article provides a summary of representative drugs and their mechanisms of action, serving as a reference for future research and experiments on MF treatment.

Contemporary medicine is primarily divided into ACE inhibitors, diuretics, and beta blockers. Examples of ACE inhibitors are perindopril and captopril; examples of diuretics are spironolactone and torsemide; and common beta-blocker medications include metoprolol and propranolol. Salvia miltiorrhiza, Astragalus membranaceus, and Angelica sinensis are the three main categories of traditional Chinese medicine. The active substances extracted from Salvia miltiorrhiza include tanshinone IIA and salvianolic acid B. Astragalus, which contain active substances such as astragaloside IV and total astragalus saponins. Angelica is known for its medicinal properties, and Angelica and Angelica polysaccharides are commonly used for its treatment.

## 2 Modern medicine

### 2.1 Angiotensin-converting enzyme inhibitors (ACEI)

Angiotensin II is a crucial factor in MF development. Increased angiotensin II activity can result in myocardial cell deficiency, hypertrophy, and inflammation, ultimately leading to myocardial fibrosis and cardiac remodeling (19, 20). Research has demonstrated that ACEIs can reduce myocardial fibrosis by blocking the generation of angiotensin II (21). Perindopril, a third-generation ACE inhibitor, has been extensively studied for its ability to reduce angiotensin I (ATI) activity by inhibiting ACE, thereby slowing the conversion of angiotensin II (ATII) (22, 23). By lowering the collagen volume fraction (CVF) and decreasing the protein levels of Col I and Col III, perindopril effectively decreased myocardial fibrosis in a rat model of diabetic cardiomyopathy (24). In a rat model of heart failure, perindopril was shown to decrease the levels of brain natriuretic peptide (BNP), COL I, and COL III, improve inflammatory cell infiltration, reduce collagen fibers, and ameliorate MF (25). In contrast, captopril, the most widely used ACE inhibitor, blocks the renin‒angiotensin system and prevents the conversion of ATI into ATII (26–29). In MI models, captopril has been shown to prevent the transformation of cardiac fibroblasts via the TGF-β1/Smad3 pathway. This action results in decreased collagen accumulation, enhanced extracellular matrix (ECM), and improved myocardial function (30). Captopril reduces ATII levels, thereby slowing the development of fibrous tissue and inhibiting collagen build-up, which ultimately results in an improvement in myocardial fibrosis (31).

The therapeutic target of ACE inhibitors is the cardiovascular system (32). However, they may lead to side effects such as hypotension (33), hyperkalemia (34), intestinal angioedema (35), and angioedema (36). However, the specific mechanism by which perindopril improves MF remains unclear (37).

### 2.2 Diuretics

Aldosterone, which is generated in the adrenal gland, significantly contributes to the progression of MF (19, 38). The activation of both the mineralocorticoid receptor (MR) and glucocorticoid receptor (GR) can induce MF and promote the differentiation of cardiac fibroblasts. Furthermore, aldosterone indirectly plays a role in the progression of MF by amplifying cardiomyocyte inflammation and inhibiting the expression of antifibrotic factors (39). Spironolactone, which acts as a mineralocorticoid receptor (MR) antagonist, reduces matrix metallopeptidase-2 (MMP-2), inhibits collagen production, and improves MF, ultimately reducing cardiac pre- and post-load and protecting the heart (40–43). Spironolactone has been demonstrated to reduce COL-I levels and collagen deposition in individuals with heart failure and preserved ejection fraction (HFpEF), resulting in the inhibition of MF (44). In contrast, torsemide, a widely used potent loop diuretic, inhibits aldosterone synthase (CYP11B2), reduces connective tissue growth factor (CTGF), and inhibits collagen accumulation, ultimately improving MF (45, 46). In rat models of heart failure, torsemide has been shown to upregulate gap junction proteins, enhance cardiomyocyte interactions, decrease myocardial collagen accumulation, improve MF, and prevent cardiac remodeling (47, 48).

Diuretics increase water and sodium excretion in the body, reduce fluid load, and improve the clinical symptoms of various diseases (49). However, they can lead to electrolyte disorders such as hyponatremia, hypokalemia, hyperkalemia, hypomagnesemia, and hyperuricemia (50). Spironolactone may have anti-androgenic side effects, but its mechanism of treating MF remains unclear (51, 52). Torsemide, which has poor water solubility, lacks a clear mechanism for MF (47, 53).

### 2.3 Beta-blockers

β-adrenergic receptors (β-ARs) can initially preserve cardiac function, but prolonged stimulation leads to the activation of cardiac fibroblasts, resulting in collagen accumulation and eventual MF (54). β-blockers are essential drugs for the treatment of cardiovascular diseases. In acute psychological failure, β-blockers can slow the resting heart rate, increase the filling pressure, and improve the survival rate of patients. In chronic heart failure, left ventricular function can be improved, thereby reducing the morbidity and mortality of patients (55–57). β-blockers can prevent renin‒angiotensin‒aldosterone system activation, sympathetic nerve activation, oxidative stress, inflammation, and other potential cardiac hazards, reduce myocardial fibrosis, improve myocardial pathological status, and prevent myocardial remodeling (58). Metoprolol, a beta-blocker, inhibits beta-adrenergic energy and reduces the levels of fibrotic adipocytokines produced by atrial adipose tissue (EAT). This inhibition suppresses cardiac fibroblast activity, decreases collagen accumulation, and improves MF (59, 60). Metoprolol decreased myocardial collagen deposition and alleviated MF (61). Propranolol, a non-selective beta-blocker, inhibits beta-adrenergic receptors, thereby neutralizing the effects of epinephrine and norepinephrine (62, 63). Propranolol also decreases fibroblast growth factor 23 (FGF-23) activity, inhibits myofibroblast function, reduces collagen accumulation, and ameliorates MF (64, 65). Propranolol reduces collagen build-up and enhances MF by regulating the TGF-β1/Smad signaling pathway (66). Nebilolol acts as a selective beta-1 adrenergic blocker and has beneficial effects on the central and peripheral vascular systems (67, 68). Nebiprolol reduces the collagen fiber area and alleviates MF by regulating caspase-3, eNOS, iNOS, and TNF-α (69).

The prognosis is poor when beta-blockers are administered to elderly patients with preserved ejection fraction heart failure (HFpEF) (70). Individuals with diabetes are more likely to experience adverse events while taking beta blockers (71). However, the precise mechanism of action of metoprolol in MF remains uncertain (58). Although propranolol is generally considered safe, it may lead to side effects, including hypoglycemia, hypotension, bradycardia, bronchospasm, and impairment of cardiovascular or respiratory function (72). Nebilolol may be associated with adverse drug events (73).

### 2.4 Other modern medicines

Other drugs, such as empagliflozin and atorvastatin, have been shown to be effective in treating MF (74). Empagliflozin reduces reactive oxygen species (ROS), decreases myocardial oxidative stress, and improves MF (75). Similarly, atorvastatin reduces myofibroblast content and MF by inhibiting oxidative stress (76).

### 2.5 Combined treatment

The combined use of drugs has a greater impact than the use of a single drug (77). When an angiotensin receptor neprilysin inhibitor (ARNI) is used in conjunction with an ACEI, it diminishes myocardial fibrosis by reducing TGF-β1 expression (78). Furthermore, the concurrent use of ivabradine HCl and trimetazidine decreased TGF-β1 and COL-L levels, resulting in decreased myocardial fibrosis (79) (Table 1).

**Table 1:** Modern medicine.

	Classification	Name	Related mechanism indicator	Reference
Modern medicine	ACEI	Perindopril	Decrease CVF, Col I and Col III	Liu et al. (24)
			Decrease BNP, COL I, COL III	Liu et al. (25)
		Captopril	Avert TGF-β1/Smad3 pathway	Wang et al. (30)
			Inhibite ATII, fibrous tissue collagen	Zhang et al. (31)
	Diuretics	Spironolactone	Reduces MMP-2,collagen production	Wang et al. (40); Kobayashi et al. (41); Sacharczuk et al. (42); Chen et al. (43)
			Reduce COL-I, collagen	Ravassa et al. (44)
		Torsemide	Inhibits CYP11B2,CTGF and collagen accumulation	Sandré et al. (45); Adam et al. (46)
			Enhance cardiomyocyte interaction, decrease myocardial collagen accumulation	López et al. (47); Watanabe et al. (48)
	Beta-blockers	Metoprolol	Suppresses cardiac fibroblast activity and collagen accumulation	Robert et al. (59); Dai et al. (60)
			Decreased myocardial collagen deposition	Liu et al. (61)
		Propranolol	Decreases FGF-23 and myofibroblast function, and collagen accumulation	Li et al. (64); Tsai et al. (65)
			Regulating TGF-β1/Smad pathway	Li et al. (66)
		Nebiprolol	Regulating caspase-3, eNOS, iNOS, and TNF-α	Mohamed and Kassem (69)
	Other modern medicine	Empagliflozin	Reducing reactive oxygen species (ROS), and myocardial oxidative stress	Wang et al. (75)
		Atorvastatin	Inhibiting oxidative stress	Song et al. (76)
	Combined treatment	ARNI conjunction with ACEI	Reduce TGF-β1 pathway	Liu et al. (78)
		Ivabradine HCl and trimetazidine	Decreases TGF-β1 and COL-L concentrations	Ma et al. (79)

## 3 Chinese medicine

### 3.1 Salvia miltiorrhiza

Salvia miltiorrhiza, a plant first documented in “Shenlong Materia Medica”, belongs to the Sage family of Lamiaceae (80). In China, Salvia miltiorrhiza is mainly used to treat angina pectoris, hyperlipidemia, and coronary heart disease and can also enhance human immunity (81). Tanshinone IIA (Tan-IIA) is a lipophilic active component of Salvia miltiorrhiza that inhibits fibrosis (82, 83). Tan-IIA inhibits fibroblast proliferation, reduces COL I and COL III accumulation, and mitigates MF (84, 85). In patients experiencing MI, Tan-IIA opposes the impact of TGF-β1 on heart fibroblasts, resulting in reduced concentrations of COL I and COL III and the mitigation of MF (86). Salvianolic acid B (Sal B), the main bioactive component of salvianolic acid, has the chemical formula C36H30O16 (87). It has been shown to be effective in suppressing fibroblast growth, lowering the levels of COL I and COL III, and improving fibrosis (88, 89). Sal B inhibits cardiac fibroblast (CF) growth, decreases collagen accumulation, and improves MF in diabetic mice by regulating TGF-β1/Smad7 expression (90). Danshen extract can significantly reduce the biochemical indices of patients with CHD, reduce the incidence of CHD, and thus protect the heart (91).

Tan IIA has a slow dissolution rate and low bioavailability, which hinders its clinical utility (85). Although its mechanism of action in treating MF remains incompletely understood (92), salvianolic acid is recognized as the most crucial active monomer component of Salvia miltiorrhiza. However, it targets only a single therapeutic pathway and does not align with the “holistic concept” in traditional Chinese medicine (93). Sal B is the most water-soluble active ingredient in Salvia miltiorrhiza; however, the precise mechanism for preventing and treating MF remains unclear (94).

### 3.2 Astragalus membranaceus

Astragalus membranaceus (AR) is a dry root obtained from the leguminous plant Bge. var. Mongolicus (Bge.) Hisao, and Astragalus membranaceus (Fisch.) Bge (95). AR is often used to regulate human immunity and cardiovascular diseases (96). It contains saponins, flavonoids, isoflavones, glycosides, flavonoids, polysaccharides, rosewood, and other active ingredients (97). Methyl glycosides, total saponins, and polysaccharides have been shown to effectively inhibit myocardial fibrosis (98). Astragaloside IV (AS-IV) is the main active ingredient (99). Astragaloside can reduce collagen I and III, inhibit oxidative stress and the p53 signaling pathway, and reduce MF (100). Moreover, AS-IV can reduce the content of COL-I and COL-III, collagen accumulation, and MI by reducing the activity of the ROS/caspase-1/GSDMD signaling pathway (101). Astragalus total saponin (ATS), the basic bioactive substance of astragalus, can reduce collagen deposition and MF by inhibiting the expression of tumor necrosis factor α and Fas ligands (102, 103). Astragalus polysaccharide is a water-soluble heteropolysaccharide (104). Astragalus polysaccharide can counteract myocardial injury, regulate the TLR-4/NF-kBp65 signaling pathway, reduce the inflammatory response, and improve MF (105). Astragalus injections are used in patients with coronary heart disease to reduce cardiovascular risk factors and protect the heart (106).

After oral administration of AS-IV, its bioavailability is relatively low, restricting its usefulness in clinical settings. Additional investigations are needed to improve the MF (101, 107, 108). The biological mechanism of astragaloside IV (AST) in MF treatment remains unclear (103). Clinical trials have only been conducted in China and have not been conducted outside the country (106).

### 3.3 Angelica sinensis

Initially, reported in “Shenlong Materia Medica”, Angelica sinensis is effective in treating cardiovascular diseases (109). Current pharmacological research has indicated that Angelica sinensis comprises a range of active constituents, such as phthalates, monoterpenes, sesquiterpenes, aromatic compounds, aliphatic hydrocarbons, derivatives, polysaccharides, and organic acids. Polysaccharides have demonstrated promising efficacy in the treatment of fibrosis (110). In an x-ray-induced MF rat model, the P13K/AKT/mTOR pathway reduced the accumulation of collagen fibers, lowered the content of COL-I and COL-III, and mitigated MF (111). Furthermore, in a study involving a rat model of myocardial infarction, Angelica sinensis inhibited macrophage proliferation, decreased TGF-β1 expression, prevented collagen deposition, and reduced myocardial fibrosis (112). Furthermore, in a hypertensive rat model, Angelica polysaccharide (ASP) mitigated MF by reducing oxidative stress, decreasing reactive oxygen species (ROS) accumulation, inhibiting cardiac fibroblast proliferation, and reducing collagen fiber accumulation (113). ASP inhibits ROS production in a dose-dependent manner, thereby reducing oxidative stress and alleviating MF (114).

Angelica sinensis, a Chinese herbal medicine, is commonly incorporated into formulas to increase medicinal efficacy (115). However, the specific mechanism by which Angelica sinensis treats MF remains unclear (111). Although ASP has significant cardioprotective properties, its specific mechanism for treating MF warrants further investigation (113, 116).

### 3.4 Other traditional Chinese medicines

Chinese medicines such as puerarin (117), triptolide (118) and ginsenoside (119) can also treat MF. Puerarin hinders the activity of heart fibroblasts, reduces the levels of COL-I and COL-III, and alleviates myocardial fibrosis by adjusting the HMGB1/TLR4-NF-kB pathway (120). Triptolide, the active compound found in Tripterygium wilfordii, decreases the number of collagen fibers, specifically COL-I and COL-III fibers, by inhibiting the Wnt/β-catenin pathway (β-catenin/c-myc/Cyclin D1). This leads to a reduction in cardiac fibroblast differentiation and alleviates myocardial fibrosis (121). The active compound RH4 in ginsenosides diminishes COL-I and COL-III content, decreases collagen accumulation, and alleviates MF by inhibiting the STAT3 and p38/MAPK signaling pathways (122) (Table 2).

**Table 2:** Chinese medicine.

	Classification	Name	Related mechanism indicator	Reference
Chinese medicine	Salvia miltiorrhiza	Tanshinone IIA	Inhibit fibroblast proliferation, COL I and COL III	Shan et al. (84); Bi et al. (85)
			Reduce TGF-β1 pathway, COL I and COL III	Qiao et al. (86)
		Salvianolic acid B	Suppresse fibroblast growth, COL I and COL III	He et al. (88); Chong et al. (89)
			Inhibit CF, collagen, TGF-β1/Smad7	Luo et al. (90)
		Danshen extract	Reduce the biochemical indices	Liu et al. (91)
	Astragalus membranaceus	Astragaloside IV	Reduce COL I and COL III, oxidative stress and p53	Shi et al. (100)
			Decrease COL-I and COL-III, ROS/Caspase-1/GSDMD	Zhang et al. (101)
		Astragalus total saponins	Reduced collagen deposition	Xiao et al. (103)
		Astragalus total saponin	Inhibiting the expression of tumor necrosis factor α and Fas ligands	Xiao et al. (103)
		Astragalus polysaccharide	Regulate the TLR-4/NF-kBp65 signaling pathway	Liu et al. (105)
		Astragalus injections	Reduce cardiovascular risk factors	Yu et al. (106)
	Angelica Sinensis	Angelica Sinensis	Reduced P13K/AKT/mTOR,collagen fibers,COL-I and COL-III	Ren et al. (111)
			Inhibited macrophages, TGF-β1,deposition of collagen	Zhao et al. (112)
		Angelica polysaccharide	Reducing oxidative stress, ROS, cardiac fibroblast, collagen fiber	Song et al. (113)
			Demonstrated ROS, oxidative stress	Pan et al. (114)
	Other Active Substances	Puerarin	Hinders heart fibroblasts, COL-I and COL-III, and HMGB1/TLR4-NF-kB path	Ni et al. (120)
		Triptolide	Decrease collagen fibers, COL-I and COL-III, β-catenin/c-myc/Cyclin D1,cardiac fibroblast	Zhang et al. (121)
		Ginsenosides	Diminish COL-I and COL-III, collagen accumulation, STAT3 and p38/MAPK pathways	Wang et al. (122)

## 4 Proprietary Chinese medicine

### 4.1 Qiliqiangxin capsule (QLQX)

Qiliqiangxin capsule, a Chinese herbal compound, is extracted from 11 different Chinese herbs, including astragalus and ginseng (123, 124). It is included in the Pharmacopoeia of the People's Republic of China and is commonly used to treat chronic heart failure (CHF) (125, 126). In a rat model of heart failure, QLQX reduced the collagen content in myocardial tissue by regulating the miR133a-endoplasmic reticulum stress-inositol-requiring enzyme 1/X-box binding protein 1 (miR133a-IRE1/XBP1) pathway (127). In a rat model of myocardial infarction, QLQX reduced type II and III collagen content, regulated collagen homeostasis, improved cardiac function, and alleviated MF (126). QLQX improves clinical symptoms and protects cardiac function in patients with chronic heart failure (123). QLQX can protect the heart by improving the clinical symptoms of patients with chronic heart failure and the levels of 6-min walking distance (6-MWD), brain natriuretic peptide (BNP), and N-terminal brain natriuretic peptide precursor (NT-proBNP) (128).

QLQX can reduce fibrosis; however, further studies and clinical trials are needed to support these findings (129). QLQX has not been fully explored for signaling pathways related to ventricular remodeling, and more high-quality RCTs are needed to improve the credibility of the evidence (128).

### 4.2 Shenqi yiqi dropping pills (QSYQ)

QSYQ is a traditional Chinese medicine (TCM). It is formed by Astragalus membranaceus Fisch. ex Bunge, Salvia miltiorrhiza Bge., Panax notoginseng (Burk.) F. H. Chen and Dalbergia odorifera T. Chen, which can be used to treat various heart diseases (130, 131). QSYQ can inhibit cardiomyocyte apoptosis, reduce type I and III collagen content, improve myocardial collagen metabolism, and reduce MF (132). QSYQ can inhibit TGF-β1, reduce type I and type III collagen, relieve myocardial collagen, and improve MF (133). QSYQ reduces extracellular matrix deposition and improves MF by regulating TGF-β1 (134). In clinical trials, QSYQ was shown to regulate the 6-minute walking distance, BNP level, and left ventricular ejection fraction in patients with ischemic heart failure (IHF), protect heart function, and improve patients’ quality of life (135). The data collected by Meta revealed that QSYQ can improve the clinical symptoms of heart failure patients with preserved ejection fraction (HFpEF), increase the 6-minute walking distance, reduce BNP, and achieve cardiac protection (136).

Although there are many pathways for the treatment of MF via QSYQ, the underlying mechanism of action requires further elucidation (132). In randomized controlled trials, the application of the QSYQ in traditional Chinese dialectical thinking has limitations (135). There is a lack of large-scale, multi-center, randomized, double-blind, and high-quality studies (137).

### 4.3 Tongxinluo capsule (TXL)

Tongxinluo capsules constitute an innovative Chinese medicine composed of 12 types of Chinese medicines, such as ginseng (138). TXL is often used to treat angina pectoris in patients with coronary heart disease (139). TXL can improve MF in the following four ways: (1) it inhibits the transition of endothelial cells to mesenchymal cells (EndMTs), activates the neuregulin-1/epithelial growth factor receptor 4-protein kinase B/protein kinase B (NRG-1/ErbB-PI3K/AKT) signaling pathway, inhibits type I and III collagen, reduces extracellular matrix deposition, and alleviates MF (140). (2) The PI3K/AKT signaling pathway is activated to reduce MF (141). (3) Inhibiting the TGF-β1 pathway, reducing collagen fiber accumulation, and improving MF (142); (4) Stress on the ventricular wall related to the MF should be reduced, the MF should be improved, the myocardium should be protected, and myocardial ischemia should be improved (143). In chronic coronary syndrome (CCS), TXL can effectively improve clinical symptoms and protect the heart (144).

The mechanism of action of TXL in improving MF is unclear, and further experimental studies are needed to determine whether it is accomplished by a single component or multiple compounds (142). Most clinical trial data on TXL are from China, and high-quality, large-scale, multi-center, and randomized controlled clinical trials are lacking (145).

### 4.4 Other proprietary Chinese medicines

Wenxin granules, Yixinshu capsules (YXS), Qifu yixin prescription (QFYX), and other proprietary Chinese medicines can also improve MF. Wenxin granules can improve MF, ventricular remodeling, and cardiac function by regulating the unfolded protein response (146). YXS regulates the retinoblastoma/histone deacetylase 1/GATA-binding protein 4 (RB/HDAC1/GATA4) pathway, improves MF, and restores cardiac function (147). QFYX improves MF and inhibits myocardial hypertrophy through the β-arrestin2 (β-arr2) pathway (Table 3) (148).

**Table 3:** Proprietary Chinese medicine.

	Classification	Related mechanism indicator	Reference
Proprietary Chinese Medicine	Qiliqiangxin capsule	Regulating the miR133a-IRE1/XBP1 pathway	Ji et al. (127)
		Reduced type II and III collagen content, regulated collagen homeostasis	Sun et al. (126)
		Improves clinical symptoms	Zhu et al. (123)
		Improving 6-MWD, BNP,NT-proBNP	Xing et al. (128)
	Shenqi Yiqi Dropping Pills	Inhibit cardiomyocyte apoptosis, reduce type I and III collagen content	Lv et al. (132)
		Inhibit TGF-β1, reduce type I and type III collagen	Lv et al. (133)
		Reduces extracellular matrix deposition andregulating TGF-β1	Lu et al. (134)
		Regulate the 6 min walking distance, BNP, and left ventricular ejection fraction	Mao et al. (135)
		Increase the 6 min walking distance, reduce BNP, and achieve cardiac protection	Wang et al. (30)
	Tongxinluo capsule	Activates NRG-1/ErbB-PI3K/AKT signaling pathway	Yin et al. (140)
		The PI3K/AKT signaling pathway	Wei et al. (141)
		Inhibiting the TGF-β1 pathway	Wang et al. (142)
		The stress on the ventricular wall related	Li (143)
		Improve clinical symptoms	Chenhao et al. (144)
	Wenxin	Regulating the unfolded protein response	Liu et al. (146)
	Yixinshu capsule	Regulates RB/HDAC1/GATA4 pathway	Zhang et al. (147)
	Qifu yixin prescription	The β-arr2 pathway	Wang et al. (148)

## 5 Conclusions and prospects

MF is the pathological basis of most cardiovascular diseases and is often closely related to myocardial infarction, myocardial ischemia, sudden cardiac death, and other cardiovascular diseases (149). Modern medicine is effective and fast, but it has potential side effects, such as hypotension and hyperkalemia (33, 34, 150). TCM can be used to treat patients according to their clinical symptoms and improve their quality of life. Owing to the advantages of multiple components, multiple approaches, and multiple targets, Chinese medicine has made progress in the study of MF; however, it also has limitations (151). For example, the cell model lacks a complex microenvironment and cannot completely replicate the pathogenesis *in vivo*; (2) the animal experimental period is long, has a high cost, and species differentiation; (3) Chinese medicine involves a variety of ingredients, making it difficult to clarify their mechanism of action; and (4) there is a lack of unified quality control standards and herbal standardization. In the analysis of TCM clinical trials, most studies were published in Chinese, the subjects were Chinese, and there were no overseas clinical studies.

Although the treatment of MF is challenging, the advantages of traditional Chinese medicine, such as good clinical efficacy, few toxic side effects, and low drug resistance, can become the focus of the treatment of myocardial fibrosis and a new research field. Therefore, TCM treatment of MF has broad research prospects. The promotion of Chinese medical treatment for myocardial fibrosis is more standardized. (1) Depending on the research purpose and experimental conditions, an appropriate method can be chosen to establish animal and cell models. (2) The relevant therapeutic drugs and mechanisms of action of traditional Chinese medicine in the treatment of myocardial fibrosis should be thoroughly and systematically explored, and the signaling pathways and core targets of this medicine should be understood. (3) As Chinese medicine has multiple components and targets, it is necessary to clarify the chemical composition of Chinese medicinal materials and compound preparations and establish a unified drug quality control standard. (4) The sample size should be increased to conduct large-scale, multi-center, randomized, double-blind, and high-quality controlled clinical trials on TCM for the treatment of MF. (5) Due to the popularity of traditional Chinese medicine in Western countries, its effects on populations in other countries should be observed to reduce sample bias. (6) The basic theories of traditional Chinese medicine should be combined with those of Western medicine and modern biological science. New traditional Chinese medicine compounds should be researched, and their clinical application should be actively promoted.
